# Dicarbonyl/L-xylulose reductase (DCXR) producing xylitol regulates egg retention through osmolality control in *Caenorhabditis elegans*

**DOI:** 10.1080/19768354.2022.2126886

**Published:** 2022-10-06

**Authors:** Yuh-Nam Kim, Seung Hyun Kim, Le Tho Son, Joohong Ahnn, Sun-Kyung Lee

**Affiliations:** a Department of Life Science and the Research Institute for Natural Sciences, Hanyang University, Seoul, Republic of Korea; b College of Forestry Biotechnology, Vietnam National University of Forestry, Hanoi, Vietnam

**Keywords:** Dicarbonyl/L-xylulose reductase (DCXR), dhs-21, xylitol, osmoregulation, egg retention

## Abstract

To support life, the osmolality of the cellular fluid is tightly regulated by various means, including osmolyte control. Dicarbonyl/L-xylulose reductase (DCXR) is a highly conserved enzyme reducing L-xylulose to xylitol, which serves as an effective osmolyte in various mammalian and human tissues such as lung epithelium, sperm, and lens. DHS-21 is the only DCXR ortholog in *Caenorhabditis elegans*, and DCXR null mutant worms accumulate eggs in the uterus. However, it has been unknown how and why the mutant worms impair egg retention. In this study, we tested whether the egg-retention in *dhs-21 (jh129*), the DCXR null mutant worm*,* is sensitive to changes in osmolarity. Low osmolality reverted the egg retention phenotype of *dhs-21(jh129)*, while high osmolarity aggravated it. Also, knock-down of either one of *osr-1*, *osm-7,* or *osm-11*, osmoregulatory genes, also rescued egg-retention phenotypes of the null mutants. The study indicates that DCXR functions in fluid homeostasis by regulating cellular osmolality in *C. elegans* and provides insights into DCXR-involved clinical conditions, such as congenital cataracts and malfunctioning lung and kidney.

## Introduction

Fluid homeostasis, which maintains the osmolality of fluid within a narrow range, is crucial in general metabolism and various cellular processes in almost all organisms. When cells encounter osmotic change, they quickly adjust their intracellular osmolality by controlling osmolyte concentration and water flux. For example, generating plasma membrane-impermeable metabolites in cytosol change the intracellular osmolality and thereby can lead to water influx. This can increase the turgor pressure of the cells, which are densely packed and organized in tissues and organs, and thereby affect the mechanical and physical properties of cells, such as elasticity, rigidity, mobility, and morphology. Therefore, cells utilize various strategies to control the proper concentration of cytosolic osmolytes to regulate cellular osmolality.

Dicarbonyl/L-xylulose reductase (DCXR) is a highly conserved enzyme reducing L-xylulose to xylitol, which serves as an effective osmolyte in animals including humans (Lee et al. 2013; Ebert et al. 2015). Xylitol shows low membrane permeability and regulates osmolality in pulmonary epithelium where DCXR is expressed (Zabner et al. 2000; Nakagawa et al. 2002). Young rats fed with a high-xylose diet accumulate xylitol in their lens and develop cataractous eyes through the glucuronic acid pathway, in which DCXR participates (Goode et al. 1996). Excessive hydration and the osmotic effect of xylitol are also implicated in congenital and infantile cataracts, in which xylitol is detected in the lens of affected children (Sulochana et al. 1997). DCXR synthesized by epididymal tissues is concentrated on the surface of human sperm, and the loss of the enzyme is associated with male infertility (Ebert et al. 2015). Osmoregulation in the epididymis is crucial for sperm maturation, and DCXR may support favorable epididymal osmolar conditions for the production of competent sperm (Johnson et al. 2011). The DCXR on the surface of sperm may directly provide osmotic protection for sperm when they are cryopreserved (Desrosiers et al. 2006). These implications strongly suggest that DCXR is actively involved in osmoregulation in animal tissues, but its role in the control of osmolality has not been directly tested at the whole organismal level.

Animal model systems utilizing versatile genetic tools have been employed to study molecular and cellular mechanisms underlying pathologic conditions associated with DCXR activity. The transgenic mice overexpressing DCXR accumulate less dicarbonyls (DCs) under surgically-induced carbonyl stress that causes nephritis, while DCXR knock-out mice are more vulnerable to protein damage in diacetyl-induced cytotoxicity (Odani et al. 2008; Hubbs et al. 2016). Our previous study using *C.elegans* DCXR null mutant, *dhs-21(jh129),* reveals that the highly conserved DCXR is required for normal fertility, egg retention, and longevity (Son le et al. 2011). While it is clear that DCXR is responsible for various phenotypes at the organismal level, it has not been explored how the lack of DCXR activity leads to a certain phenotype.

*C. elegans* hermaphrodites usually hold one row of eggs in their uterus with an average of approximately 15 under normal culture conditions. However, worms can hold more eggs in case their egg-laying behavior is not properly regulated (Schafer 2005). Also, avid worms cultured at high osmolality conditions retain eggs almost twice as many as a normal condition. In this study, we asked a question about whether the egg retention phenotype shown in DCXR null mutant worms, *dhs-21(jh129),* is caused by low cellular osmolality induced by the probable low level of xylitol due to the lack of DCXR activity. We found that severe egg-retention in *dhs-21(jh129)* was relieved when the mutant worms were cultured at low osmolality conditions. In addition, the knock-down of osmotic regulatory genes, *osr-1*, *osm-7*, and *osm-11*, in DCXR mutants also rescued egg retention. This study indicates that the egg-retention in *dhs-21(jh129)* is likely caused by low osmolality, presumably due to low cellular xylitol level, and provides a piece of experimental evidence supporting the conserved osmotic regulatory function by DCXR.

## Materials and methods

### Strains and growth conditions

Worms were maintained at 20 ℃ on nematode growth medium (NGM) seeded with *Escherichia coli* OP50 strain. Strains used in this study were: wild-type (N2 Bristol), *dhs-21(jh129)*, *dhs-21(jh129)[pdhs-21::dhs-21::GFP]* and *N2[pdhs-21::dhs-21::GFP]*. N2 was obtained from CGC (Caenorhabditis Genetics Center) at the University of Minnesota (St. Paul, MN, USA). *dhs-21(jh129)* mutant worm was isolated from TMP/UV mutagenized library (Son le et al. 2011). *dhs-21* rescue line, *dhs-21(jh129)[pdhs-21::dhs-21::GFP]*, and *dhs-21* over-expression line, *N2[pdhs-21::dhs-21::GFP],* were obtained by injecting 2027bp *dhs-21* promoter+1.8 kb full *dhs-21* genomic sequence into *dhs-21(jh129)* and *N2* respectively (Son le et al. 2011).

### Osmotic stress assays

All osmotic stress assays were carried out at 20 ℃. NGM plates containing 0, 34.2, 100, or 400 mM NaCl were prepared. Adult hermaphrodite worms were placed on NGM plates and then removed after being allowed to lay eggs for 3 h. After hatched and cultured, young adult worms were placed on osmotic stress assay plates for two days and then examined for egg retention in the uterus.

### RNAi

To construct feeding RNAi plasmids, cDNA fragments of *osr-1, osm-7,* and *osm-11* were amplified using these primers: *osr-1*, 5’-AAT ACT GCA GAT GAG GAT GAA AGT GCA TTC-3’ (forward primer, F) and 5’-CGT AAA GCT TCT TTG CAT CAA TCA TTT CAG-3’ (reverse primer, R); *osm-7*, 5’-ATG CCC ATG GAT GTC CGA AAT ACG AGC GCT-3’ (F) and 5’-AAA ACT GCA GTT GAG CGC CCG CCA CAA TTC-3’ (R); *osm-11*, 5’-AAA ACT GCA GAT GAA CTT TAT TAC CGT CGC-3’ (F) and 5’-ATG CAA GCT TTT AAT ACT GCA CTG GAG TGA-3’ (R). Each fragment was cloned into L4440 vector and then transformed into *E.coli* strain HT115. 20 microliter of saturated bacterial culture of each feeding RNAi clone was spread onto assay plates containing 400 μm IPTG to induce RNAi.

## Results

### *dhs-21 (jh129)* mutants retain eggs and are sensitive to external salt concentration.

**Figure 1.**
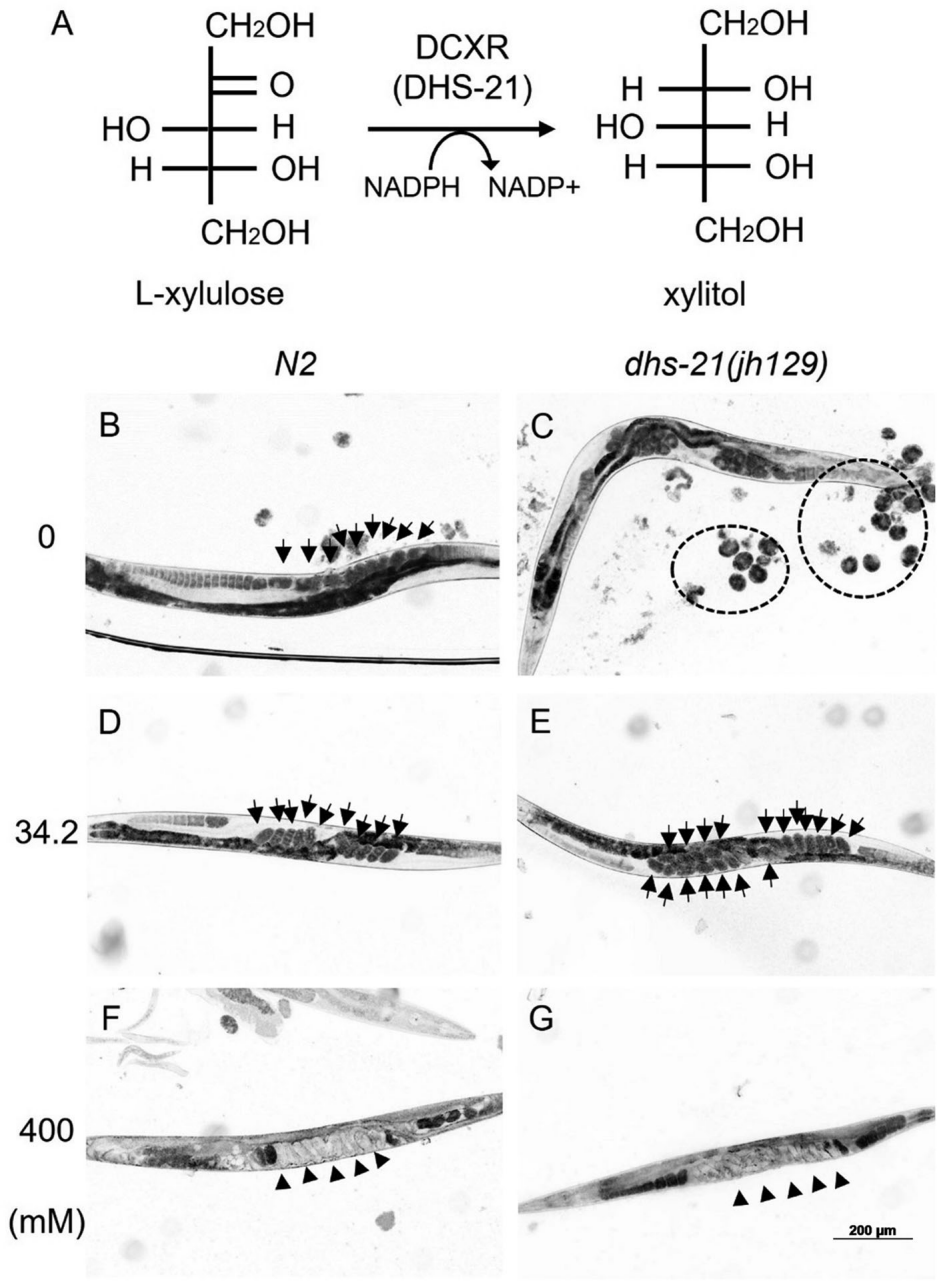
Egg-retention in *dhs-21 (jh129)*, which is lack of active DCXR. (A) DHS-21, *C. elegans* DCXR, reduces L-xylulose to make xylitol using a cofactor NADPH. Egg-retention phenotype in *dhs-21 (jh129)* is sensitive to external concentration of NaCl. *dhs-21 (jh129)* at 34.2 mM NaCl (E) holds more eggs (arrows) in its uterus than the wild type, *N2* (D). At 0 mM NaCl (B and C), many *dhs-21 (jh129)* worms release retained eggs responding to 40 μM levamisole, but most *N2* worms did not. At 400 mM NaCl (F and G), both N2 and *dhs-21 (jh129)* have embryos (arrowheads) that stay in the uterus for an extended period, overgrow and hatch inside the uterus.

In our previous study, we reported that *dhs-21* is expressed in uterine seam (utse) cells, the spermathecal-uterus (sp-ut) valve, intestine, gonadal sheath cells, and spermatids, and *dhs-21(jh129)* mutants, which do not produce DHS-21 proteins, have reduced brood size, decreased lifespan, and egg-retention in their uterus (Son le et al. 2011). To examine the possibility that the egg-retention in the mutant is induced by the change in cellular osmolality, we first cultured the mutants at different osmotic conditions and then observed egg-retention status. *dhs-21(jh129)* mutants cultured on NGM plates containing 34.2 mM NaCl had much more eggs in the uterus than wild types as expected (Figure 1D and E). The mutant worms cultured on 0 mM NaCl plates were placed on an agar pad with a drop of 40 μM levamisole, which was added to paralyze the worms. Many of these worms laid eggs on the agar pad because eggs were pushed out of the uterus through the vulva due to contraction induced by levamisole (Figure 1C). However, wild-type worms did not respond much to the added levamisole (Figure 1B). These results indicate that there is a difference between wild type and *dhs-21(jh129)* worms in reaction to the low osmolality culture condition. When cultured at 400 mM NaCl, both wild-type and *dhs-21(jh129)* mutant worms were extremely stressed by high osmolality and showed extreme egg-retention, which eventually caused a bag-of-worms and subsequent death (Figure 1F and G). Therefore, we hypothesized that *dhs-21(jh129)* mutant has a low level of the osmolyte xylitol; thus cellular osmolality in the mutant may be lower than *N2*, and in a low osmotic environment, *dhs-21(jh129)* may release pressure generated by osmotic stress associated with low cellular osmolality in the mutant worms.

### Low external osmolality rescues the egg-retention phenotype of *dhs-21(jh129)*.

**Figure 2.**
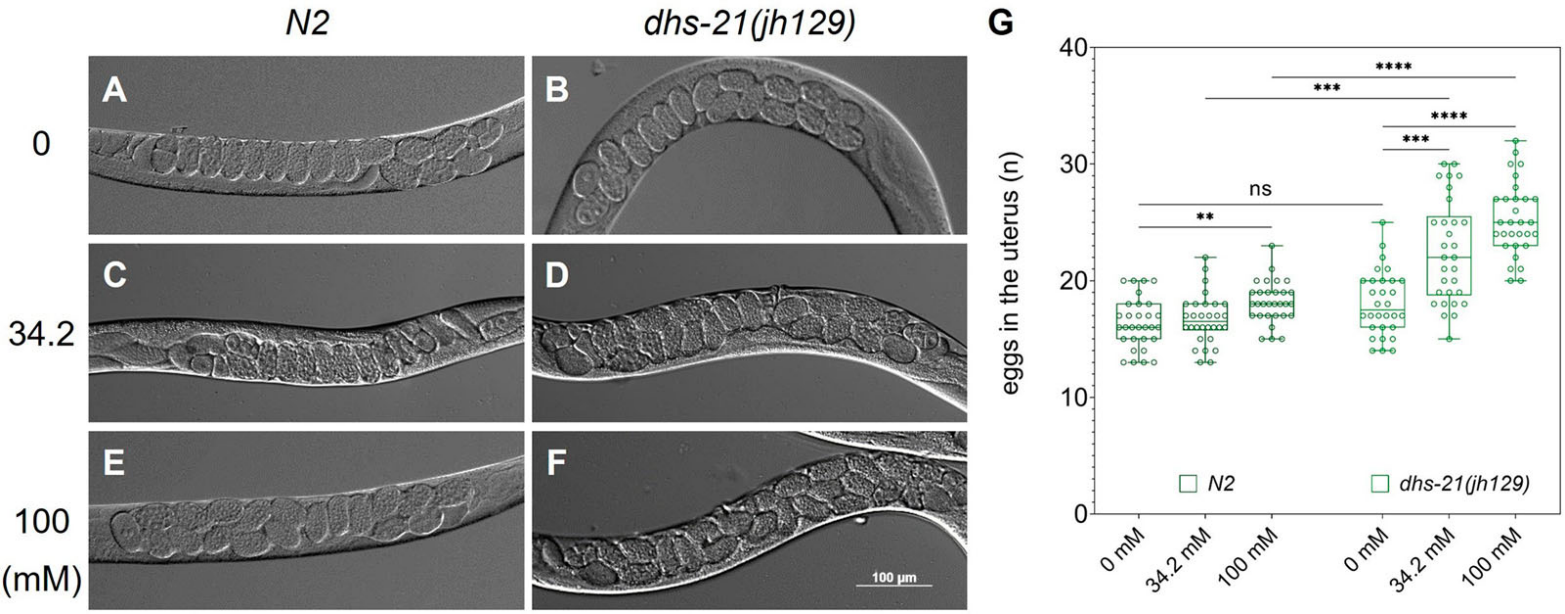
Egg-retention in *dhs-21 (jh129)* is relieved by low osmolality. (A and B) *dhs-21 (jh129)* cultured in 0 mM NaCl for two days reduces the number of eggs in the uterus to a level comparable to *N2*. *N2* and *dhs-21 (jh129)* (C and D) and 100 mM (E and F) are shown. *dhs-21 (jh129)* (D) holds more eggs than *N2* (C) at 34.2 mM, and the egg-retention in the mutant is aggravated at 100 mM (E and F). **p < 10^−4^, ***p < 10^−6^, ****p < 10^−10^, *t*-test.

To test the hypothesis, we cultured young adult worms of both wild type and *dhs-21(jh129)* in NGM plates containing 0, 34.2, and 100 mM NaCl for two days (Figure 2). Most worms were viable in these conditions, which allowed further examination of egg retention in the uterus. To avoid a burst of egg-laying shown in Figure 1, we used 500 mM sodium azide instead of levamisole to immobilize worms for microscopy. The egg-retention shown in *dhs-21(jh129)* was significantly reduced when cultured on the plate with 0 mM NaCl, but wild-type animals showed little difference (Figure 2A-D, and G). Culturing worms at 100 mM NaCl significantly worsened egg retention, while *dhs-21(jh129)* exhibited severer egg-retention than wild-type (Figure 2E-G). These results suggest that the internal osmolality of *dhs-21(jh129)* should be lower than wild-type, thus more sensitive to the change in external osmolality.

### RNAi to *osr-1*, *osm-7,* or *osm-11*, which develops resistance to osmotic stress, relieves egg-retention phenotype.

**Figure 3.**
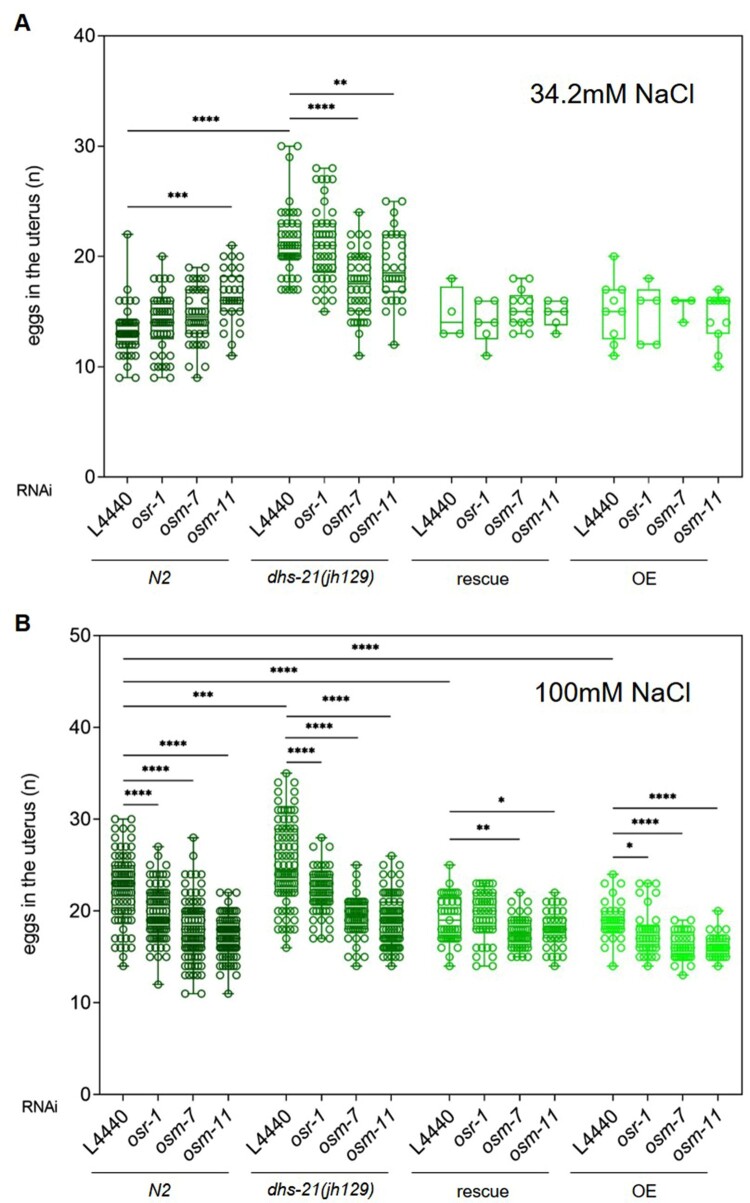
RNAi to *osr-1*, *osm-7* or *osm-11* rescues egg-retention in *dhs-21 (jh129)*. *N2*, *dhs-21 (jh129),* rescue line (rescue, *dhs-21(jh129)[pdhs-21::dhs-21::GFP]*) and overexpression line (OE, *N2[pdhs-21::dhs-21::GFP]*) are cultured at 34.2 mM (A) or 100 mM (B) of NaCl, being treated with feeding RNAi to *osr-1*, *osm-7* or *osm-11*, and the numbers of eggs in the uterus are scored. *p < 0.05, **p < 0.01, ***p < 0.0001, ***p < 0.00001, *t*-test.

*C. elegans* adapt to osmotic stress by increasing the in-body concentration of glycerol that serves as an effective osmolyte (Wheeler and Thomas 2006). *osr-1* is a regulator of the osmotic stress response (Solomon et al. 2004). *osm-7* and *osm-11* are members of the osmotic stress resistance family (osm), which are required for normal avoidance of high salt concentration and share the Dos-motif, which functions in Notch receptor signaling (Komatsu et al. 2008; Singh et al. 2011). Worms having mutations in either one of these genes exhibit high glycerol levels, which presumably lead to resistance to osmotic stress (Wheeler and Thomas 2006). Having higher glycerol levels, *osm-7* or *osm-11* mutant worms develop higher resistance to osmotic stress than *osr-1* mutant worms (Wheeler and Thomas 2006). To see whether the egg retention phenotype of *dhs-21* mutant is modulated by *osr-1*, *osm-7,* or *osm-11*, we treated *N2*, *dhs-21(jh129), dhs-21 rescue,* and *dhs-21 overexpression lines* with feeding RNAi against *osr-1, osm-7,* or *osm-11* at 34.2 and 100 mM NaCl conditions*.* Our feeding RNAi was effective because RNAi-treated worms were all resistant to high osmotic stress (Supplementary Data 1). At 34.2 mM NaCl, we found that *osr-1* RNAi did not significantly change the number of in-uterus embryos in *dhs-21(jh129)* whereas *osm-7* or *osm-11* RNAi did (Figure 3A). In contrast, at 100 mM NaCl, all the numbers of embryos retained in the uterus of *dhs-21(jh129)* mutant worms were significantly decreased by RNAi to all three genes (Figure 3B). In the case of *N2*, *dhs-21 rescue,* and *dhs-21 overexpression lines*, at 34.2 mM NaCl, RNAi treatment to each of the three genes did not much affect the number of embryos in the uterus, except *N2* treated with *osm-11* RNAi treatment, which exerted the strongest effect (Figure 3A). It is likely that both *dhs-21* rescue or *dhs-21*-overexpression line express *dhs-21* at high level, not to be further affected by RNAi to *osr-1*, *osm-7* or *osm-11*. At 100 mM NaCl, which causes modest osmotic stress and makes worms retain eggs, the RNAi treatments developing resistance to osmotic stress relieve egg-retention in all types of worms, especially RNAi to *osm-7* or *osm-11*, the strongest effects (Figure 3B). Altogether, these results indicate that *dhs-21(jh129)* mutant worms are under low internal osmolality conditions, presumably due to the low xylitol level caused by the lack of DCXR/DHS-21, which can be reversed by RNAi to *osr-1*, *osm-7* or *osm-11,* which confers osmotic resistance with high glycerol concentration.

## Discussion

Living organisms properly maintain fluid homeostasis by adjusting osmolyte concentrations in their cells. Reducing L-xylulose, DCXR generates xylitol which is not freely cell-membrane permeable, thus plays a role of active osmolyte in human tissue (Zabner et al. 2000). Imbalanced cellular osmolarity can interfere with proper mechanical and physical properties of cells, which in turn experience physiological dysfunctions. In this study, we found that abnormal osmolarity plays a role in egg retention phenotype observed in free-living soil nematode *C.elegans* lacking the sole DCXR. Low extracellular osmolality or knock-down of osmotic regulatory genes, *osr-1*, *osm-7*, or *osm-11,* rescued the egg-retention phenotype of the DCXR null mutant worms. Therefore, DCXR has a conserved function as an osmolyte, actively participating in the maintenance of proper osmolality required for egg retention, a critical reproductive activity.

*C. elegans* is a versatile nematode model system utilizing powerful genetic tools and adapting various advanced engineering and simulation techniques (Levine and Lee 2020; Cho et al. 2021). *C. elegans* hermaphrodites self-fertilize and accumulate fertilized eggs in their uterus. Adult worms usually hold 10∼15 eggs at any given time. The eggs in the uterus are laid through the vulva which consists of specialized muscles that are tightly controlled by a set of specialized neurons (Schafer 2005). In case the egg-laying activity is inhibited, worms tend to retain eggs in their uterus. For example, egg retention phenotype is often observed in the worms defective in egg-laying muscles and neurons. External conditions also can influence and inhibit the activity of the egg-laying machinery, and hypertonic environmental condition strongly inhibits egg-laying and causes egg retention in worms. In the previous study, we reported that the egg retention phenotype observed in *dhs-21* mutant is not due to abnormalities in the vulva and HSN egg-laying neural circuit (Son le et al. 2011). DCXR/DHS-21 is highly expressed in some reproductive organ tissues that are critical in egg-retention phenotype. Together with our findings in this study, the egg-retention in *dhs-21(jh129)* mutants may not be attributed to defective egg-laying apparatus but likely to imbalanced osmolality in reproductive organs, resulting from loss of enzyme producing an effective osmolyte, xylitol.

DCXR/DHS-21 is highly expressed in uterine seam cells, gonadal sheath cells, and spermathecal uterine valve, all of which are critical in gamete production, ovulation, fertilization, and egg-laying (Son le et al. 2011). Uterine seam cells attach the uterus to the lateral epidermal seam cells to hold the uterus in place (Lints R. and Hall D.H. 2009a). Gonadal sheath cells surround a syncytium of germ cells and generate signals controlling ovulation (Lints R. and Hall D.H 2009b). Fertilized oocytes in the spermatheca pass the spermathecal-uterine valve to enter the uterus (Lints R. and Hall D.H 2009b). All of these reproductive tissues are highly membraneous and consist of the syncytium, probably adaptive to osmolality change to support their required high elastic resilience. Therefore, a high level of DCXR/DHS-21 may contribute to the high capacity of osmolality regulation in these specialized tissues, which exhibit highly elastic and flexible features such as passing through oocytes, fertilized eggs, or embryos.

L-xylulose is produced in a glucuronate pathway, which accounts for some part of glucose catabolism, and then converted to xylitol by DCXR (Meng et al. 2016). A person with pentosuria caused by DCXR deficiency excretes about 2∼4 g of L-xylulose (Lasker and Enklewitz 1933). Because DCXR is concentrated only in some specific tissues such as liver, kidney, and testis, the conversion of L-xylulose to xylitol in these specific tissues is predicted to be higher than in nearby tissues with low DCXR expression, thus likely to significantly contribute to osmolality regulation.

Different organisms use widely different osmolytes, and many compatible osmolytes are metabolites that do not disturb internal metabolic function (Yancey et al. 1982). Such metabolite glycerol is a type of polyols, which are common cellular osmolytes in various species from yeast and alga to animals and plants (Yancey et al. 1982; Hohmann 2015). Responding to osmotic stress, these organisms increase glycerol content by increasing glycerol synthesis or uptake. At this end, *C.elegans* increases the expression of glycerol-3-phosphate dehydrogenases and phosphoglycolate phosphatase, all of which produce glycerol (Wheeler and Thomas 2006; Possik et al. 2022). In this study, we show that expression change in DCXR/DHS-21, an enzyme generating osmolyte xylitol, contributes to osmolality control in worms. Therefore, the regulation of effective osmolyte-synthesizing enzymes plays a key role in osmoregulation.

**Figure 4.**
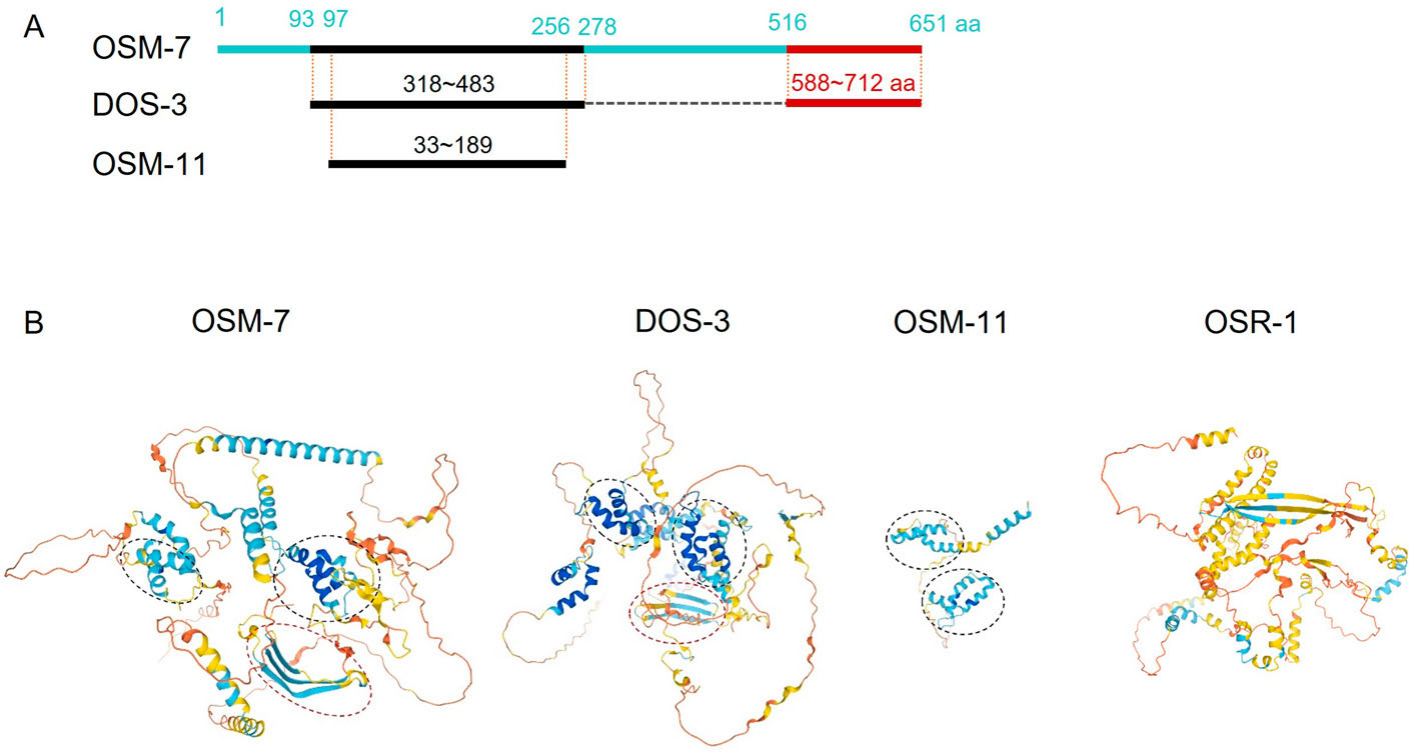
*C. elegans* novel protein OSR-1 and the Dos-like proteins, OSM-7, OSM-11, and DOS-3. (A) Schematic alignment of the Dos-like proteins. BLAST search indicates the Dos-like motifs that are homologous to each other between OSM-7, OSM-11, and DOS-3. OSM-7 isoform A consists of 651 amino acids, DOS-3 does 712 amino acids, and OSM-11, 189 amino acids. DOS-3 is never reported to be osmotic resistant and has not been tested in this study. (B) Predicted 3D structures are generated by AlphaFold, using full-length sequences of each protein (Jumper et al. 2021; Varadi et al. 2022). α-helical regions surrounded with black-dotted lines and β-pleated sheets with red ones are the regions of black lines and of red lines in (A), respectively. Both OSM-7 and DOS-3 have a transmembrane stretch at their N-terminal region (Komatsu et al. 2008). The predicted structure of the novel protein OSR-1 is different from the Dos-like proteins.

To adapt to osmotic changes in the environment, various cells in diverse organisms regulate the osmoadaptive signaling pathways such as mitogen-activated protein kinases (MAPKs) and Notch signaling and synthesize compatible osmolytes such as glycerol (Yancey et al. 1982; Komatsu et al. 2008; Singh et al. 2011; Hohmann 2015; Possik et al. 2022). In *C. elegans*, a novel gene *osr-1* genetically interacts with a p38 MAPK pathway to adapt to osmotic stress (Solomon et al. 2004). Both *osm-7* and *osm-11* are the conserved Dos (Delta and osm-11-like)-motif protein coligands acting with DSL (Delta, Serrate, and LAG-2) ligands that activate Notch receptor signaling, which is required for development and neuro-behaviors (Singh et al. 2011) (Figure 4). Loss of *osm-7* and *osm-11* causes adaptation to hyperosmotic stress, independent from MAPK or PKC pathways. Interestingly, basal glycerol levels are highly elevated in the worms having mutations in *osr-1*, *osm-7*, and *osm-11*, in which *gpdh-1* gene, a glycerol-producing enzyme, is highly upregulated (Wheeler and Thomas 2006). Therefore, the osmoadaptive strategies regulating certain cell signaling pathways and producing effective osmolytes are also conserved in nematodes. RNAi treatment to *osr-1*, *osm-7*, or *osm-11*, rescues the egg retention phenotype of *dhs-21(jh129)* mutant worms, in which cellular osmolality is expected to be low. The glycerol contents in *osm-7* and *osm-11* mutant worms resistant to osmotic stress are the largest among the tested *Osr* mutants, approximately five times as much as *osr-1* mutant (Wheeler and Thomas 2006). These results indicate that the egg-retention phenotype shown in *dhs-21(jh129)* is in an osmolality-dependent mode. Therefore, xylitol, the product of DCXR *dhs-21*, is likely to serve as an effective osmolyte in mammals, including humans, actively participating in osmoregulation and supporting the reproductive process in *C. elegans*.

**Figure 5.**
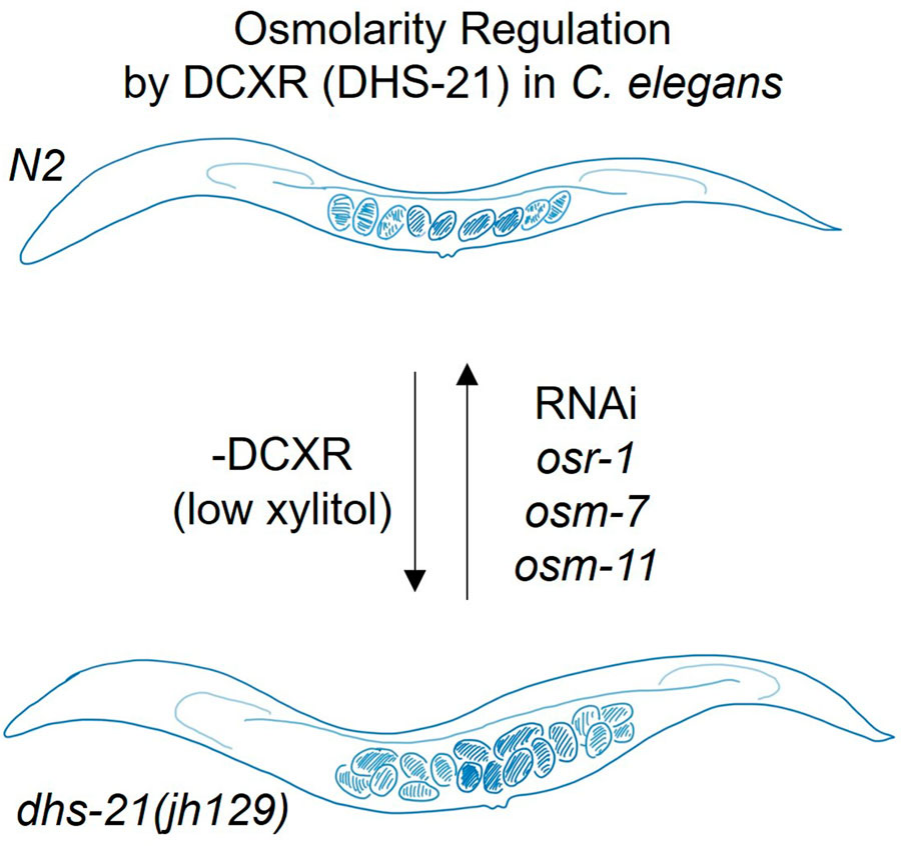
DCXR/DHS-21 regulates osmolality in *C. elegans.* Worms lacking functional DCXR/DHS-21 hold unusually many eggs in their uterus, presumably due to low internal osmolality caused by low xylitol levels. RNAi to osmoregulatory genes such as *osr-1*, *osm-7*, or *osm-11*, which contain high content of glycerol in worms, relieves excessive egg retention.

Living organisms largely rely on fluid homeostasis, which provides the proper aqueous environment for normal cellular structure and function. Adjusting the levels of compatible osmolytes is a widely-adapted osmo-stress coping strategy through diverse species. Xylitol serves as an effective osmolyte in human tissues such as lung epithelium, sperm, and lens, where the highly-conserved xylitol synthesizing DCXR is expressed. *C. elegans* lacking DCXR/DHS21 retain more eggs in their uterus than wild-type, and this egg retention phenotype is likely due to an imbalance in osmoregulation because RNAi to *osr-1*, *osm-7*, or *osm-11*, which increases osmolality by generating excess glycerol is effective to suppress the egg retention (Figure 5). Xylitol-driven osmoregulation seems to be a conserved mechanism that supports specific cellular functions in diverse animals from nematodes to humans, which express the multifunctional DCXR enzyme, which detoxifies by reducing tissue-damaging agents and osmobalances by generating xylitol, in specific tissues (Lee et al. 2013; Ebert et al. 2015).

## Supplementary Material

Supplemental Material
