# Supplementary figures and images for: Dicarbonyl/L-xylulose reductase (DCXR) producing xylitol regulates egg retention through osmolality control in Caenorhabditis elegans
Source: Anim Cells Syst (Seoul). 2022 Oct 6;26(5):223–31. doi: 10.1080/19768354.2022.2126886 (PMC9586651; doi:10.1080/19768354.2022.2126886)

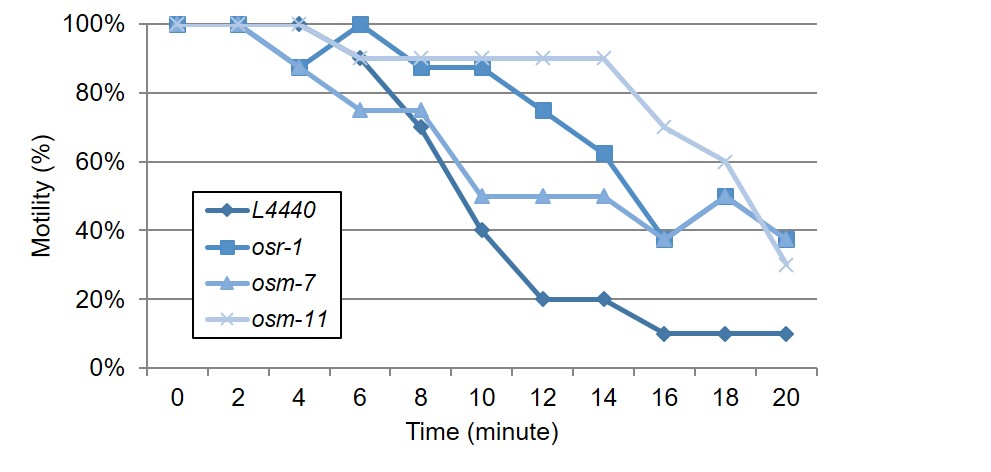

Supplement: Supplemental Material [file TACS_A_2126886_SM1000.jpg]
